# A comparison of node vaccination strategies to halt SIR epidemic spreading in real-world complex networks

**DOI:** 10.1038/s41598-022-24652-1

**Published:** 2022-12-09

**Authors:** F. Sartori, M. Turchetto, M. Bellingeri, F. Scotognella, R. Alfieri, N.-K.-K. Nguyen, T.-T. Le, Q. Nguyen, D. Cassi

**Affiliations:** 1 Dipartimento di Scienze Matematiche, Fisiche e Informatiche, Università di Parma, Via G.P. Usberti, 7/a, 43124 Parma, Italy; 2 Dipartimento di Fisica, Politecnico di Milano, Piazza Leonardo da Vinci 32, 20133 Milano, Italy; 3 Center for Nano Science and Technology@PoliMi, Istituto Italiano di Tecnologia, Via Giovanni Pascoli, 70/3, 20133 Milan, Italy; 4 INFN, Gruppo Collegato di Parma, 43124 Parma, Italy; 5 Faculty of Basic Science, Van Lang University, Ho Chi Minh City, Vietnam; 6 John Von Neumann Institute, Vietnam National University Ho Chi Minh City, Ho Chi Minh City, Vietnam; 7 Institute of Fundamental and Applied Sciences, Duy Tan University, Ho Chi Minh City, 700000 Vietnam; 8 Faculty of Natural Sciences, Duy Tan University, Da Nang City, 550000 Vietnam

**Keywords:** Complex networks, Computational science

## Abstract

We compared seven node vaccination strategies in twelve real-world complex networks. The node vaccination strategies are modeled as node removal on networks. We performed node vaccination strategies both removing nodes according to the initial network structure, i.e., non-adaptive approach, and performing partial node rank recalculation after node removal, i.e., semi-adaptive approach. To quantify the efficacy of each vaccination strategy, we used three epidemic spread indicators: the size of the largest connected component, the total number of infected at the end of the epidemic, and the maximum number of simultaneously infected individuals. We show that the best vaccination strategies in the non-adaptive and semi-adaptive approaches are different and that the best strategy also depends on the number of available vaccines. Furthermore, a partial recalculation of the node centrality increases the efficacy of the vaccination strategies by up to 80%.

## Introduction

Immunization through vaccination is an essential issue of research with obvious implications for public health^1–3^. Network science plays a fundamental role in epidemiology, and many recent pieces of research modeled disease spreading using network models^4–10^. A spreading disease can be described as a network where nodes represent the individuals and links (edges) represent the social contacts between them^9,11–13^. The two classes of interventions that can be implemented to reduce the size of an outbreak can be divided into pharmacological interventions, PI, such as vaccinations, and non-pharmacological interventions, NPI, such as social distancing, washing hands, and lockdowns. NPIs are a set of changes in social behavior that aim to reduce the social interactions (links or connections) among individuals, like during lockdowns^14,15^, or by reducing the risk that the disease is transmitted for each social interaction, like by wearing masks^16^. One of the fundamental problems in network epidemiology is finding the best vaccination strategy to halt epidemics^6,7,13,17^. Node removal (attack) on complex social networks is an optimal framework to model vaccination strategies over a population where diseases can spread^13,17–22^. As a first approach, the problem can be mapped to the classic percolation problem in graph theory, where nodes are immunized (removed) from the network to induce the fastest network dismantling^6,7,17^.

In this context, it has been shown that removing highly connected nodes is an efficient strategy for determining a fast network dismantling^6,7,17^. Nonetheless, focusing on how node removal affects the network’s connectivity is a simple and static representation of vaccination effects on the disease spread. Fine-grained models should encompass further aspects of the epidemics, such as the temporal dynamics of the disease spreading. This can be done by solving the classical susceptible-infected-recovered (SIR) model^23^ over a network^10,12,24^. Coupling SIR epidemiological models and network structure provides further information about the disease spread, such as evaluating the effect of node vaccination/removal strategies (NVS) to reduce the pace of the epidemics, the peak of infected individuals, or the total number of infected at the end of the epidemic^12^. For these reasons, understanding which node removal strategy is the most efficient for curbing SIR epidemics spreading in networks is a promising tool for developing effective vaccination strategies. In this paper, we implement seven NVSs over a set of twelve real-world networks from different fields of science. The NVSs adopted here are based on notions of node centrality from network science and graph theory. We studied two classes of NVSs, the one where the centrality of each node is calculated at the beginning of the simulations, and we refer to this class as non-adaptive, and the one where the node centrality is recalculated after a fraction *r* of nodes have been removed, as semi-adaptive.

To test the NVS efficacy, first, we analyze their ability to dismantle the network connectivity, i.e., to decrease the largest connected component (**LCC**)^25,26^. Then, we study the NVS efficacy to curb two indicators of a SIR epidemic outbreak: the total number of infected individuals at the end of the outbreak (**TI**) and the maximum number of simultaneously infected individuals, or infection peak (**IP**). A non-stochastic approach that also includes the dynamical parameters of the SIR that relies on the Dynamic Message-Passing equations (DMP) allows calculating the set of individuals that minimize the TI. We find that in case of limited vaccine availability, the best strategy depends on the topology of the network where the outbreak is evolving and on the fraction of individuals that can be vaccinated. Furthermore, we showed that using a semi-adaptive algorithm reduces the three SI up to $75\%$ for the same vaccination target. Finally, we showed that the vaccine requirement needed for the $10\%$ threshold of acceptable **TI** and **IP** is lowered from $50$ to $60\%$ if a non-random NVS is implemented.

## Results

### SIR and vaccination strategies

To understand how node vaccination strategies (NVS) in case of limited vaccine doses affect the spread of an epidemic in real-world complex networks, we considered the SIR dynamic epidemic model. SIR is a compartmental model, where every individual can be in one of three states/compartments: Susceptible to be infected **S,** Infected individual **I**, and Removed (or Recovered) from the infection dynamic **R** individuals^27^. We used the implementation of the SIR model on a network as in^9^. In this frame, each network node represents an individual, and the adjacency matrix **A** dictates which nodes are connected to whom and, therefore, how the outbreak will evolve in the network^9,12,28,29^. Two parameters regulate the dynamic of the epidemic:$\upbeta$ represents the probability that a Susceptible node connected to an Infected one will become Infected, and $\upgamma$ is the probability that an Infected node will overcome the infection becoming Recovered. Furthermore, we assume that a vaccinated individual falls in the same class as a Recovered one and cannot infect any of its neighbors.

The optimal vaccination strategy would be to vaccinate all the individuals in the network, but that is often not possible, both because of limited availability of vaccines, money, doctors, and time or due to fragile subjects that cannot be vaccinated. In this work, we aim to answer the question: what is the best vaccination strategy if only a fraction q of the population can be vaccinated?

To quantify the NVS efficacy on real-world complex networks, we analyzed three spread indicators (**SI**s) for 20 vaccination targets q. The first is the size of the network’s largest connected component (**LCC**). The **LCC** accounts for the maximum number of connected nodes in the network, and it is a commonly used quantity to evaluate the efficacy of node vaccination (removal) strategies^7,12,20^. The **LCC** is a purely topological indicator of the epidemic spreading, accounting solely for the maximum number of nodes that can be infected. The second metric we used is the total infected (**TI**) individuals, i.e., the number of infected individuals at the end of the outbreak event. If all the individuals in the network share the same risk factor, the **TI** is directly proportional to the number of hospitalizations and the total number of casualties at the end of the outbreak event. The third metric is the infected peak (**IP**), i.e., the maximum number of simultaneously infected individuals during the outbreak; **IP** is an important indicator to evaluate the possible overload of the health system, which would increase the mortality rate of both the epidemic itself and other diseases.

Finding the optimal set of nodes to vaccinate to reduce the size of the **LCC** below a threshold C is an NP-complete problem^30^. Therefore, we are following a heuristic approach by assuming that the most central node is the one whose removal minimizes the three SI most. The NVSs we are comparing in this work are seven ways to define the centrality of each node, which we listed in Table 3 in the Methods section.

**Figure 1:**
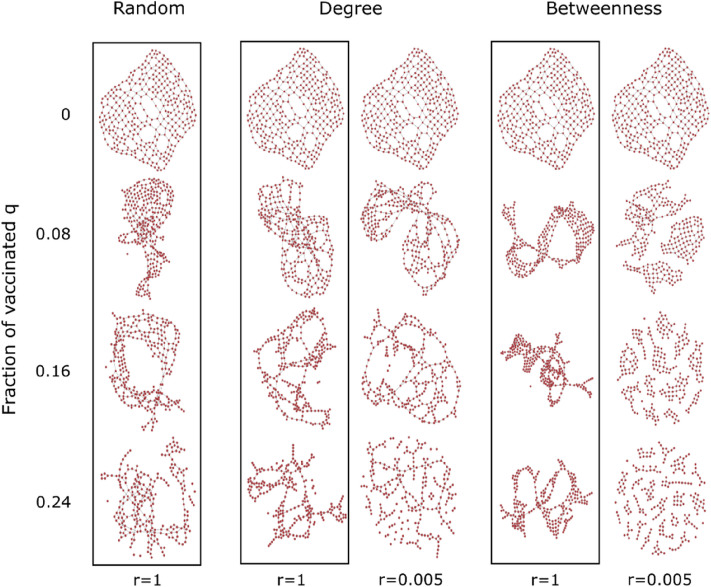
Effect of three nodes vaccination strategies for four fractions of vaccinated individuals q: 0, 0.08, 0.16, and 0.24 over the BE3 network. The boxed graphs correspond to the reduced network obtained following a non-adaptive algorithm, while non-boxed ones to the reduced network obtained from a semi-adaptive algorithm, with recalculation steps $r=0.005$.

In Fig. 1, we give a graphical representation of the outcomes of three of the NVS to the BE3 network^31^, a road network in Beijing city. In the dashed boxes, we show the reduced BE3 network after removing the fraction *q* of nodes with the highest centrality, with $q=0, 0.08, 0.16,$ and $0.24$. In Fig. 1, we see that the more nodes we remove (vaccinate), the fewer links (representing the infective social interactions) remain in the network, and, therefore, the smaller the capability of an outbreak to spread within the network. This is clearer for the semi-adaptive, $r=0.005$, that triggers a visible fragmentation of the **LCC**.

### Non-adaptive strategies

**Figure 2:**
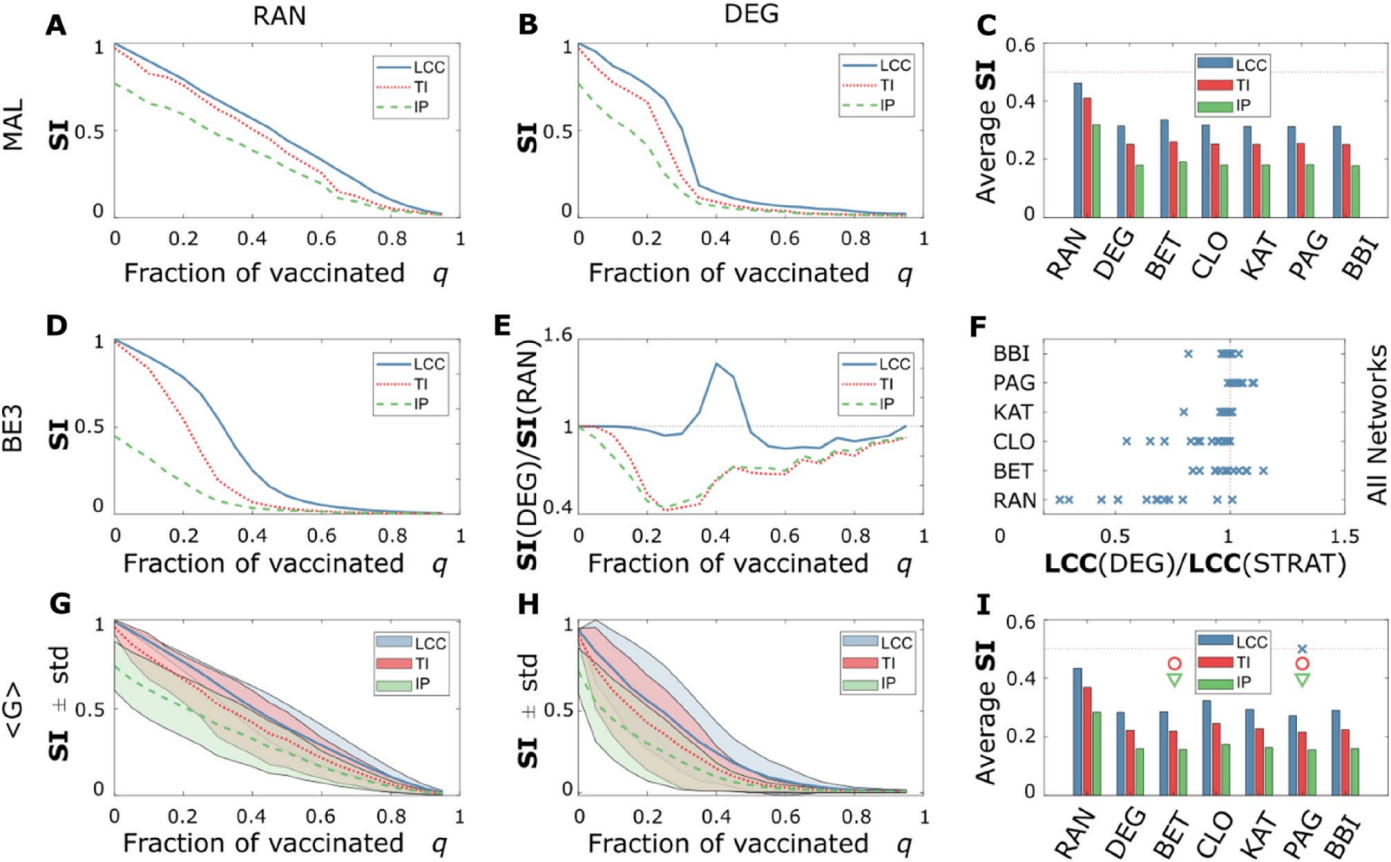
Comparison of non-adaptive strategies. (**A**, **B**) spread indicators **SI** for MAL network^50^ as a function of the fractions of vaccinated q, for the RAN and the DEG strategies, respectively; (**C**) average **SI**s, defined as the average value that the three **SI**s assume in the $20$ fractions of vaccinated q in the MAL network; (**D)** we show the dependency of the **SI**s for BE3; (**E**) **SI**s values obtained for the DEG strategy normalized by the ones obtained by the RAN strategy, a value smaller than one indicates that the DEG is more effective than RAN at minimizing that **SI**; (**F**), number of times in which the DEG strategy is more effective than the strategy on the y-axis at minimizing the **LCC**; (**G, H) SI** $\pm$ 1 σ confidence averaged over the $12$ analysed networks; (**I**) average **SI**s, defined as the average value that the three **SI**s assume in the $12$ networks and the $20$ fractions of vaccinated q. (**I)** the overlayed symbols, blue cross, red circle, and green triangle, indicate the optimal strategies to minimize the **LCC**, the **TI**, and the **IP,** respectively; in (**C**), all the NVS but RAN and BET are equally effective in minimizing the three **SI**s, namely their relative difference is smaller than $1/50$: $\left( {\frac{{{\text{STRAT}}}}{{\min \left( {{\text{SI}}\left( {{\text{STRAT}}} \right)} \right)}} - 1 < \frac{1}{50}} \right)$. The notation < G > indicates that the quantities in (**G**, **H**, **I**) are averaged over the 12 analysed networks.

The first two strategies we implemented are the *Random* strategy (RAN), where the centrality of each node is a random value between $0$ and $1$, and the *Degree* strategy (DEG), where the centrality of each node is the number of first neighbors that such node has. In Fig. 2A and B, we show how the three **SI**s decrease when the network is subjected to RAN and DEG, respectively. However, unsurprisingly DEG, which contains information about the network topology, is more effective at reducing all three SI (see Fig. 2C for a direct comparison).

The RAN strategy is not always worse than the DEG at minimizing the three **SI**s, as shown in Fig. 2 D and E. In the BE3 network, for instance, the DEG strategy is more effective at minimizing the **TI** and the **IP** but, surprisingly, not at minimizing the **LCC**. In Fig. 2 F, bottom line, we show that in the $12$ analyzed real-world networks, only BE3 has this unintuitive behavior; in the same figure, we also show how often the other five NVS are more effective than DEG at minimizing the **LCC**, while Betweenness (BET) is the one that on a specific network performs better, it shows high variability. *Pagerank* (PAG), instead, performs consistently better than DEG.

In Fig. 2G and H, we show the values of the three **SI**s averaged over the $12$ analyzed networks, and in Fig. 2I, the average score of the seven strategies averaged both over the networks and the fraction of vaccinated individual q. When considering the average over the network, the best NVS to minimize the **LCC** is PAG; the best strategies for minimizing **TI** and **IP** are PAG and BET. Interestingly the DEG efficacy, which only contains information about the number of adjacent neighbors of a node, is comparable to BET and significantly outperforms *Closeness* (CLO) in minimizing the **LCC** despite BET and CLO containing more information about the topology of the network.

### Semi-adaptive strategies

In the non-adaptive NVSs we used in Fig. 2, the centrality of each node is calculated on the initial network and is no longer updated. After each vaccination (node removal), the network structure changes, and the difference between the node centralities calculated on the initial network and the updated one increases. The difference between the degree calculated on the original network and the reduced one increases is smaller or equal to the number of removed nodes. For non-local NVS, such as the BET and the CLO, this difference varies in a non-linear way.

**Figure 3:**
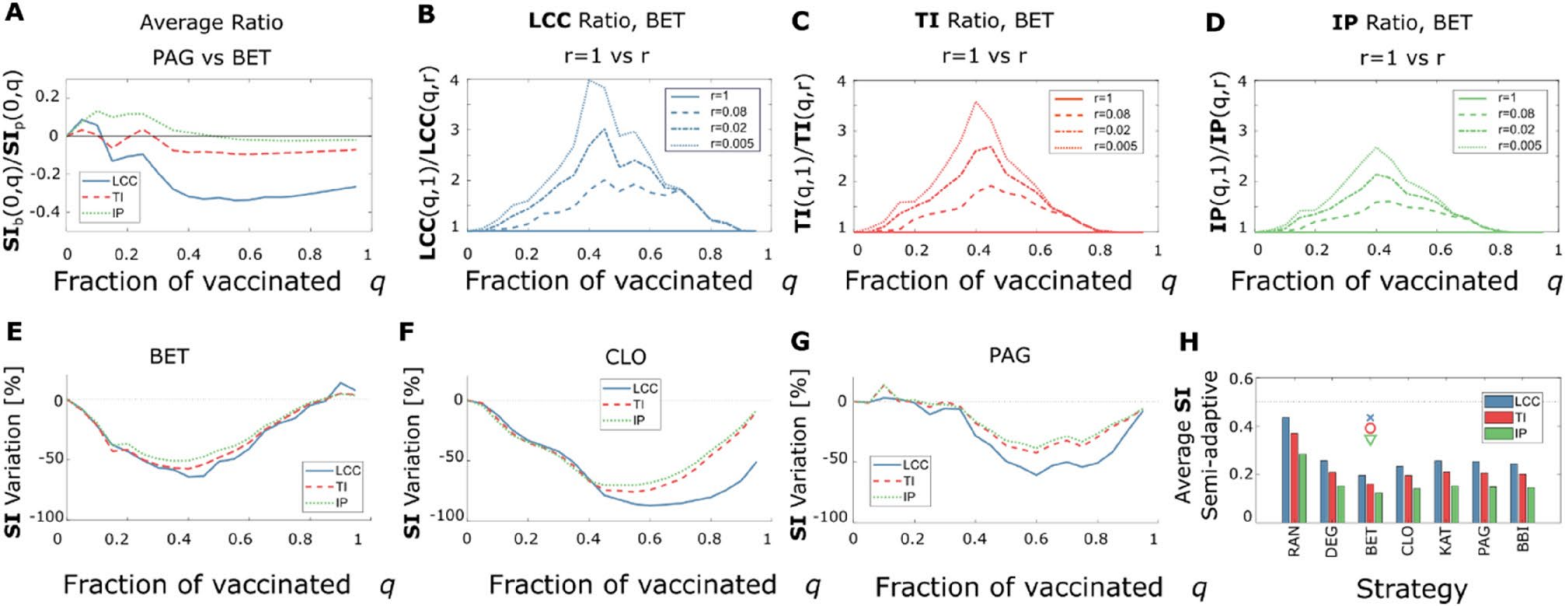
Comparison of Semi-Adaptive Strategies. In this figure, we compare the three **SI** obtained for non-adaptive strategies with the ones obtained for semi-adaptive strategies. (**A**) **SI** values averaged between 0 and q, obtained with BET and normalized by the ones obtained with PAG. A value bigger than one means that BET outperforms PAG, and a value smaller than one means that PAG outperforms BET, non-adaptive strategies; (**B**, **C**, **D)** relative decrease of **LCC**, **TI**, and **IP** respectively moving from a non-adaptive algorithm, solid line, to refined recalculation steps, dashed for $\mathrm{r}=0.08$, dot-dashed for $\mathrm{r}=0.02$, and dotted for $\mathrm{r}=0.005$; (**E**, **F**, **G**) percental reduction of the three **SI** going from a non-adaptive algorithm to a 0.005-adaptive algorithm, for three the BET, CLO, and PAG strategies respectively; (**H**) average **SI**s, defined as the average value that the three **SI**s assume in the 12 networks and the 20 fractions of vaccinated q, for a 0.005-adaptive algorithm.

In Fig. 3, we show the outcomes from semi-adaptive NVS. In Fig. 3A, we show that for low values of the fraction of vaccinated q, when the difference between the initial centralities and their real value is small, the BET strategy outperforms, on average, PAG. Therefore, we introduced a new class of algorithms that we called semi-adaptive, where after a fraction of nodes r is removed from the network, recalculate the centrality of each node. Because the number of recalculations is fixed, the algorithm complexity of such semi-adaptive strategies is the same as the non-adaptive ones. For the BET strategy, moving from a non-adaptive to a semi-adaptive strategy can reduce the **LCC**, the **TI**, and the **IP** up to a factor of $3$ to $4$, as shown in Fig. 3B, C, and D, for a recalculation factor $r=0.005$.

The consequences of implementing a semi-adaptive strategy on a single network can be seen in Fig. 1 for $r=1$ (no recalculation) and $r=0.005$. The reduced networks obtained by removing the fraction* q* of vaccinated individuals are visibly more fragmented for the same vaccination target for the semi-adaptive NVS.

In Fig. 3E, F, and G, we show the percental variation induced by going from a non-adaptive algorithm to a semi-adaptive one with $r=0.005$, of the three **SI**s averaged over the $12$ networks for three NVS. We show that CLO is the one that improves the most when implementing recalculation. When considering a recalculation factor $r=0.005$, the NVS that, on average, triggers the best minimization of the three **SI**s is BET, as shown in Fig. 3H.

**Figure 4:**
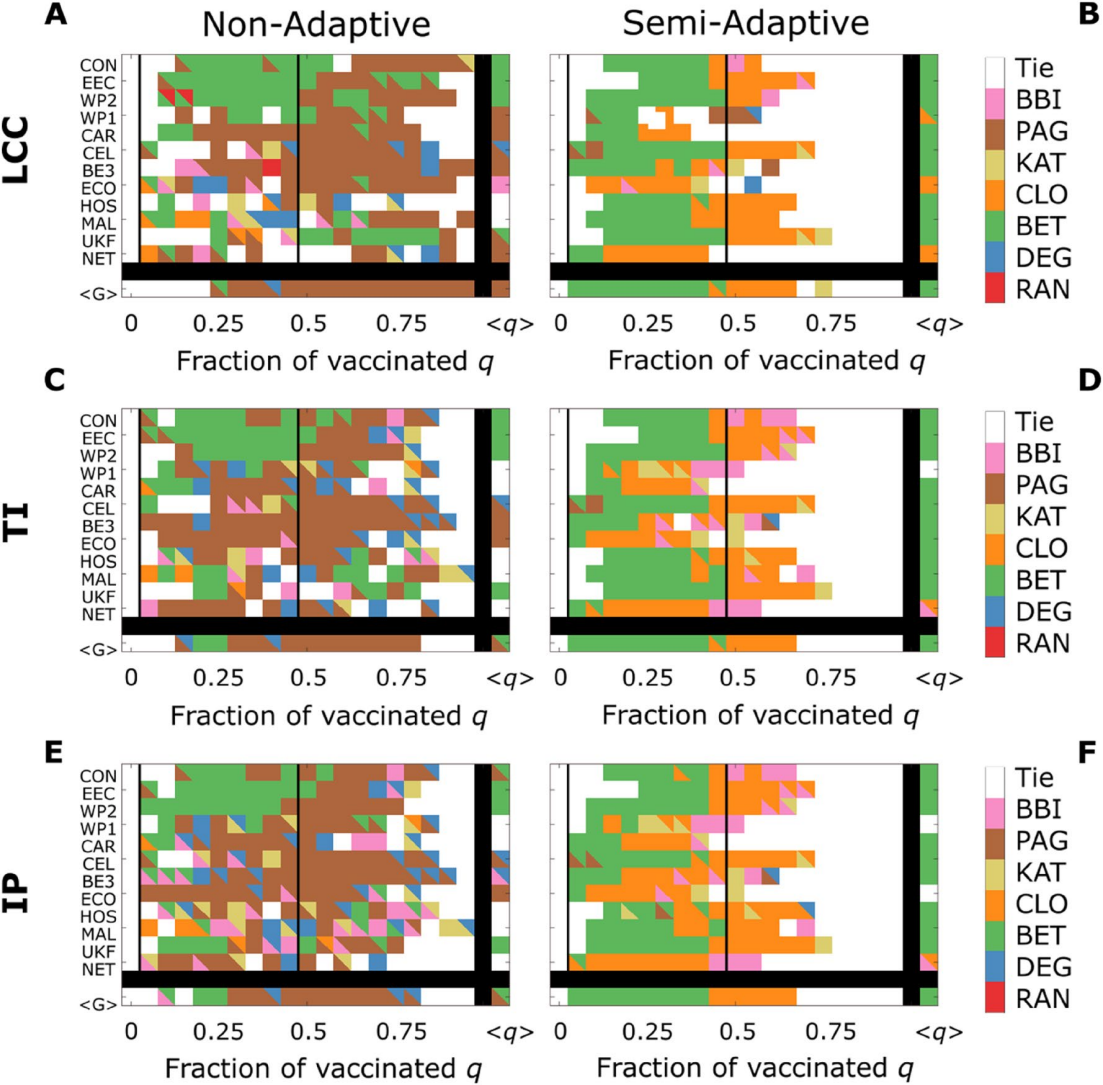
Best Strategy comparison. In this figure, we show, for both non-adaptive and semi-adaptive strategies, the best NVSs to minimize each **SI**s for the $12$ networks. (**A**, **C**, **E**), optimal strategies for the non-adaptive approach; (**B**, **D**, **F**) optimal strategies for the semi-adaptive approach with a recalculation step $r=0.005$. (A, B) best strategies to minimize **LCC**; (**C**, **D**) best strategies to minimize **TI**; (**E**, **F**) best strategies to minimize **IP**. The bottom line in each subplot shows the strategy that, on average, minimizes the SI for that value of q, while the leftmost column the strategy that, on average, minimizes the corresponding **SI** of that network. The colour code is the same in the six subpanels, a description of the acronym of the strategies can be found in Table 3, and a summary of the acronyms of the networks in Table 2; in case of a tie between two strategies, both colours are indicated, and in case of a tie between three or more strategies the colour white is used. A tie happens when the relative difference between the best NVS and the second-best one is smaller than $1/50$. The notation < G > indicates that the quantities are averaged over the $12$ analysed networks and the notation < q > indicates that the quantities are averaged over the $20$ analysed fraction of vaccinated q.

In Fig. 4A, C, and E, we show that in $5$ out of the $12$ networks, one of the three **SI**s is minimized by an additional strategy, while in 2 networks (ECO, BE3) the **SI**s are minimized by different NVS. In Fig. 4B, D, and F, we show that the difference between the efficacy of the optimal strategies is more limited in the semi-adaptive case, with $r=0.005$, in 2 out of the 12 networks (NET, WP1), two of the **Si**s are minimized by an additional strategy, and in one network (ECO) the **SI**s are minimized by two different NVS.

For $q>0.5$, in most of the semi-adaptive cases, with $r=0.005$, there are at least two NVS that perform equally. Therefore, Supplementary Table S1 shows the percentage of cases where each NVS is among the best to minimize the corresponding **SI**. For higher values of* q*, the strategy that more often minimizes the three **SI**s in the non-adaptive scenario is PAG, and in the semi-adaptive one is CLO. Interestingly, in the semi-adaptive approach, while BET is the worse of the non-random strategy in the $q>0.5$ regime, it outperforms the others in the $q<0.5$ region, and it becomes the best overall NVS when averaging across all the vaccination targets *q*.

We show a summary of the best strategies as a function of the recalculation step *r*, the **SI**, and the network in Table 1.

**Table 1:** Best vaccination strategy per network.

**SI**	r	Network name											
		CON	EEC	WP2	WP1	CAR	CEL	BE3	ECO	HOS	MAL	UKF	NET
**LCC**	1	BET 0.34	BET 0.32	BET 0.40	PAG 0.20	PAG 0.19	**DEG** **PAG** **0.36**	PAG 0.31	BBI 0.13	TIE 0.33	TIE 0.33	**BET** **PAG** **0.40**	PAG 0.08
	0.08	TIE 0.34	BET 0.30	BET 0.32	**KAT** **BBI** **0.19**	TIE 0.19	BET 0.29	DEG 0.26	BET 0.12	**BET** **PAG** **0.30**	BET 0.28	**BET** **CLO** **0.35**	**CLO** **BBI** **0.08**
	0.02	**BET** **BBI** **0.32**	BET 0.29	BET 0.27	**BET** **CLO** **0.18**	BET 0.16	BET 0.28	BET 0.19	BET 0.12	BET 0.28	BET 0.24	BET 0.28	BBI 0.07
	0.005	BET 0.30	BET 0.28	BET 0.23	**BET** **CLO** **0.18**	BET 0.14	BET 0.24	BET 0.11	CLO 0.11	BET 0.28	BET 0.24	BET 0.28	CLO 0.06
**TI**	1	BET 0.29	BET 0.27	BET 0.30	PAG 0.14	TIE 0.15	**DEG** **PAG** **0.31**	PAG 0.19	PAG 0.09	TIE 0.26	TIE 0.26	BET 0.35	TIE 0.05
	0.08	**BET** **PAG** **0.29**	BET 0.26	BET 0.24	KAT 0.13	TIE 0.14	BET 0.24	DEG 0.18	TIE 0.09	TIE 0.25	BET 0.22	BET 0.31	TIE 0.05
	0.02	BET 0.28	BET 0.25	BET 0.21	**BET** **CLO** **0.13**	BET 0.12	BET 0.23	BET 0.15	BBI 0.09	BET 0.23	BET 0.19	BET 0.26	**CLO** **BBI** **0.05**
	0.005	BET 0.26	BET 0.24	BET 0.18	TIE 0.13	BET 0.11	BET 0.20	BET 0.09	BET 0.08	BET 0.23	BET 0.19	BET 0.26	**CLO** **BBI** **0.05**
**IP**	1	**BET** **PAG** **0.23**	BET 0.22	BET 0.21	**DEG** **PAG** **0.10**	TIE 0.10	**DEG** **PAG** **0.23**	**DEG** **BBI** **0.08**	PAG 0.06	TIE 0.21	TIE 0.19	BET 0.27	TIE 0.03
	0.08	TIE 0.22	BET 0.21	BET 0.18	**KAT** **BBI** **0.09**	TIE 0.10	BET 0.19	BBI 0.07	TIE 0.06	TIE 0.20	**BET** **CLO** **0.17**	BET 0.25	TIE 0.03
	0.02	BET 0.21	BET 0.20	BET 0.16	TIE 0.09	BET 0.09	BET 0.18	BBI 0.06	BBI 0.06	BET 0.19	BET 0.15	BET 0.21	**CLO** **BBI** **0.03**
	0.005	BET 0.20	BET 0.20	BET 0.14	TIE 0.09	BET 0.08	BET 0.16	BET 0.05	TIE 0.06	BET 0.19	BET 0.15	BET 0.21	**CLO** **BBI** **0.03**

For each recalculation step r and network, we indicate the best NVS to minimize the average **SI** over q, as described in the methods. For each box we indicate the acronym of the best NVS and the value of the average of that strategy. If the relative difference between two NVS is smaller than $1/50$, we indicate both the best strategies in bold, while when the three (or more) best strategies differ by less than $1/50$, we label that case with TIE.

### Vaccination requirements

One of the main concerns of governments regarding the COVID-19 pandemic has been to minimize the total number of infected individuals and to slow down the infection rate, not to overwhelm the health systems^32^. To achieve these aims, the governments immediately implemented non-pharmacological interventions (NPIs), like enforcing social distancing and lockdowns^14,33^, and began immunizing individuals with a vaccination campaign^34^. To study this problem, we investigate what percentage of the population needs to be vaccinated to keep the **TI** and the **IP** below a certain threshold $\theta$.

**Figure 5:**
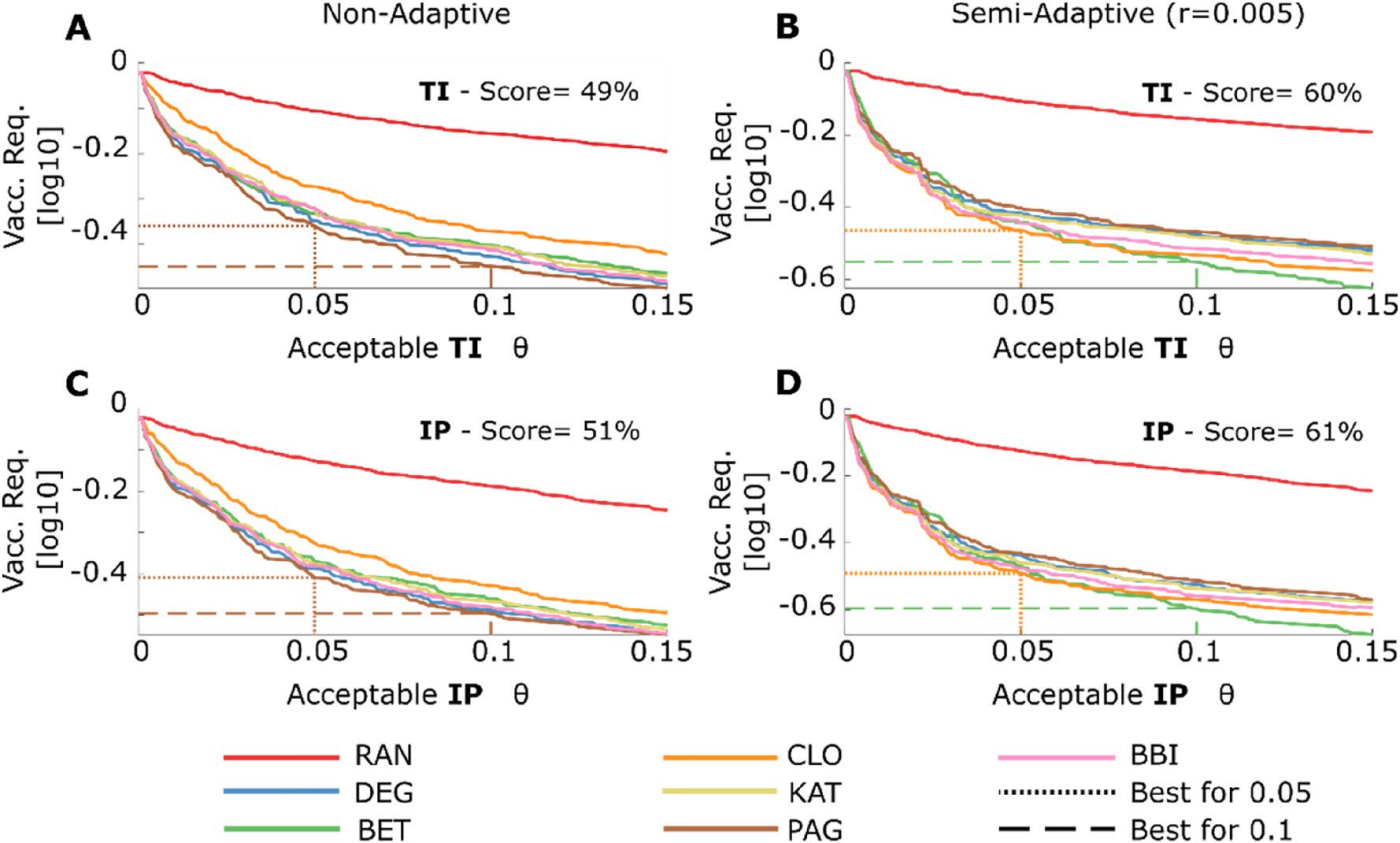
Vaccination Requirements. In this figure, we show the vaccination requirement to keep the **TI** and the **IP** below the acceptable threshold ϑ. (**A**, **C**) fraction of the population that needs to be vaccinated to keep the acceptable **SI**s value below a threshold $\uptheta$, in case of non-adaptive algorithm ($\mathrm{r}=1$); (**B**, **D**) fraction of the population that needs to be vaccinated to keep the acceptable **SI**s value below a threshold $\uptheta$ in case of semi-adaptive algorithm with a recalculation step $\mathrm{r}=0.005$ .The dashed and the dotted lines indicate the best strategy for $\uptheta =0.1$, and $\uptheta =0.05$ respectively. The score is the percental reduction in the vaccination requirement going from the random strategy (RAN) to the best one for $\uptheta =0.1$. We show the y-axis in log scale to increase the readability of the figure.

In Fig. 5, we show that the NVS minimizing the vaccination requirement is the PAG, for a non-adaptive approach, and for a semi-adaptive approach with a low acceptable **SI**s threshold, namely higher vaccination requirements, is the CLO, and for a higher acceptable threshold, is the BET. Furthermore, going from a random vaccination strategy (RAN) to a non-random one reduces by 49–61% the number of vaccines to keep the spread of the disease below the threshold $\theta =0.10$. Further, going from a non-adaptive algorithm to a semi-adaptive one reduces the vaccination requirement of the best strategy to keep the spread of the disease below the same threshold by $20\%$.

## Discussion and conclusion

This research has focused on finding an effective way to curb epidemic spreading in real-world complex networks via vaccinations. Finding the optimal set of nodes to minimize the size of the **LCC** is an NP-complete problem approached in the literature both in random networks^24,25,35^ and in real-world networks^25,36^. We followed the approach in^25^ by using established centrality measures to determine the node vaccination priority. Here we analyzed two classes of NVSs, the non-adaptive, where the centrality is calculated on the original adjacency matrix, and the semi-adaptive ones, where the centrality of each node is updated after a fraction* r* of nodes is removed. The computational complexity of a semi-adaptive NVS is the same as the non-adaptive equivalent because the centrality calculation is repeated a fixed number of times. In contrast, in the adaptive approach, where nodes rank is computed after each node removal^37^, the computational complexity increases with the number of nodes in the network.

Random NVSs are generally less effective at curbing the epidemic than a strategy containing information about the network nodes’ social structure^22,30^. We showed that the vaccination requirement to keep the **SI**s below $10\%$ of the total population dramatically benefits from a non-random NVS, saving an average of $49$ and $60\%$ of the vaccines, compared to the random NVS for the non-adaptive and the semi-adaptive approach, respectively. These outcomes give important insights into performing effective vaccination policies. On the one hand, these outcomes show that when implementing vaccination strategies, it is fundamental to consider the network’s structure. On the other hand, they would suggest that gathering information about the local structure of the network nodes, such as sampling the number of contacts of individuals, might significantly improve the efficacy of the vaccination process. This would be particularly important since many vaccination campaigns used to curb the COVID19 spreading neglected the network structure^38–40^. We explored six non-random NVSs and found that in the non-adaptive approach, both the betweenness centrality, BET, and the PageRank centrality, PAG, are the two most effective strategies for minimizing the size of the **LCC**, but also the other two spread indicators. In the semi-adaptive approach, the two best NVSs are based on the betweenness (BET) and closeness (CLO) centrality of the nodes. The former is very effective at minimizing all three **SI**s when the fraction of available vaccines is below $45\%$, while the second performs better in the $45-70\%$ range, above which their performances are comparable. These outcomes indicate that, when possible, considering the global and whole structure of the network, as required to implement the Pagerank (PAG), betweenness (BET), and closeness (CLO) strategies, it would be an optimal way to vaccinate the population.

We found that implementing a semi-adaptive (recalculated) NVS significantly reduces the vaccination requirement compared to a non-adaptive approach. This finding would indicate that when implementing vaccination policies in real-world networks, considering the changes in the network structure along the vaccination process may significantly improve the efficacy of the vaccination process to curb epidemics. Noteworthy, we improved the non-adaptive NVS by partially recalculating the node centralities. In the semi-adaptive NVS, we recalculated the centrality of each node after $0.5\%$, $2\%$, and 8% of the node were removed, and this semi-adaptive approach does not increase the complexity of the algorithms. Some strategy, like CLO, profits from moving from a non-adaptive to an adaptive approach more than others, like PAG. We attributed this different behavior to the locality of the NVS considered. We argue that highly non-local measures may experience a higher increase in performance by passing from a non-adaptive to a semi-adaptive approach. On the contrary, the more local a centrality measure is, the less it will improve by implementing a semi-adaptive NVS. This is because if the centrality measure is local, the centrality of a node will be less affected by the removal of a random node in the network. For example, CLO is defined as the inverse of the harmonic average of the distance. The distance between points is a highly non-local measure that can vary considerably by removing a single node. On the other hand, PAG quantifies a node’s authority by looking at the authority of the nodes pointing at it. Therefore, the information about distant nodes is embedded in the adjacent nodes. Therefore, using a semi-adaptive algorithm always improves the efficacy of the NVS, and it is especially beneficial in the case of non-local NVS.

The outcomes of our research show that when planning to contain an epidemic event is important to consider the social structure of interaction between individuals. Knowing the network structure underlying social interactions is necessary to calculate the centrality measures discussed in this work, but it is a promising way to perform effective vaccination strategies. Unfortunately, considering the real-world social network structure is a challenging task. Nonetheless, in the last years, several methods have been developed to reconstruct the social interaction network in metropolitan areas like Berlin^15^ and to approximate the value of some of the centrality measures having only access to local and partial information^22^.

Another problem that became evident during the current COVID pandemic was the reluctance of a large fraction to get vaccinated or to wear masks; this suggests the importance of coupling two dynamics, one for the virus and one for the vaccination-predisposition^41^. As developed in Ref.^42^, the Dynamical Message-Passing method can be used to approach these coupled dynamics.

Finally, we showed that in $5$ of the $12$ networks, the best NVS to minimize each **SI**s is the same, and in $2$ out of $12$, the best strategies to minimize the three **SI**s are different. Since the **LCC** is a static and the most straightforward indicator of the maximal possible disease spreading, it is necessary to analyze the two dynamic spread indicators **TI** and **IP**, to contain the outbreak. This is particularly important considering that many studies in network science adopted the **LCC** to evaluate the efficacy of the NVS^6,7,17,25^.

## Methods

### Simulations algorithm

To study the impact of different node vaccination strategies (NVS) on the dynamic of an epidemic outbreak, we used the following protocol: (1) We import one of the $12$ real-world networks; (2) We infect one random node in the network; (3) We set a vaccination target q, namely the fraction of the network nodes that will be vaccinated; (4) We remove Nq nodes from the network; (5) We simulate on the reduced network a SIR dynamic, and for each step, we track the number of nodes in the three populations: S, I, and R. (6)We stop the simulation when no more nodes are in the state I.

### Real-world networks summary

In this work, we analyzed twelve real-world networks from different fields. In Table 2 below, for each network, we summarized the type, the number of nodes, the number of links, the average node degree, and the network’s diameter. We implement the SIR over each network’s largest connected component (**LCC**). Four real-world networks (CON, WP1, WP2, and HOS) are face-to-face networks, where the interaction between individuals and the interaction time are accounted for^43^. We consider only the interactions that happened in the first $12$ h for these networks.

**Table 2:** Real-world networks summary.

ID	Network name	Acronym	Type	N	E	< k >	⌀	Reference
1	Conference	CON	Social	257	3248	25.3	6	^43^
2	Email EU Core	EEC	Email	986	25,552	51. 8	7	^44^
3	Workplace 2	WP2	Social	159	2180	27.4	7	^43^
4	Workplace 1	WP1	Social	47	138	5.9	7	^45^
5	Cargoship BB	CAR	Ship	821	4342	10.5	9	^46^
6	C-Elegans	CEL	Neural	297	2345	15.8	5	^47^
7	Beijing 3	BE3	Street	322	1075	6.7	27	^31^
8	E-Coli	ECO	Protein	1100	3637	6.6	11	^48^
9	Hospital	HOS	Social	43	358	16. 7	5	^49^
10	Malawi	MAL	Social	84	346	8.2	5	^50^
11	UK Faculty	UKF	Social	81	817	20.2	4	^51^
12	Netscience	NET	Citation	397	914	4.8	17	^52^

Real-world networks used in this research with acronym, network type, reference, and basic statistical features: number of nodes (N), number of edges (E), average node degree (< k >), and network diameter (⌀).

The epidemic SIR-dynamic we considered in this work, strictly speaking, can be properly applied to face-to-face networks. Nonetheless, coupling node removal and SIR-like dynamic could be useful to describe a variety of real problems in other types of networks, such as halting computer viruses or stopping epidemic spreading among airports or cities. For example, vaccinating vertices in CAR, and BE3, could be interpreted as a localized lockdown to prevent the spread of an epidemic in a geographical area and a district, respectively. For this reason, we also considered a variety of real-world networks that are not face-to-face.

### Non-adaptive approach to vaccination

In the non-adaptive approach of the NVS, we rank the nodes according to the node centrality metrics at the beginning of the simulation (before any node removal), and we do not update the rank. The non-adaptive node removal (attack) strategies are also named ‘initial node attack’ or ‘not-recalculated node attack’^25,53,54^. In the case of ties, i.e., nodes with equal centrality rank value, one of the nodes with the highest centrality nodes are removed at random.

### Semi-adaptive approach to vaccination

In the semi-adaptive approach of the NVS, the node’s rank is updated during the removal process. First, we calculate the rank of each node, and after a fraction $r<q$ of nodes is removed, the rank of each node is calculated in the reduced network. We repeat this procedure until we vaccinate a fraction* q* of the nodes. In the last iteration, we vaccinate a fraction of nodes smaller than *r* to vaccinate exactly a fraction *q* of the population. In this work, we considered four values of* r*: $r=0.005$, $r=0.02$, $r=0.08$, and $r=1$.

### Node vaccination strategies (NVS)

In this work, we implemented seven NVSs; except for the random vaccination (RAN), the others require knowing the entire adjacency matrix of the network.

#### Random

The first strategy we approach is to assign to each node a random weight. This vaccination strategy has the significant advantage of not requiring prior knowledge of the network’s topology.

#### Degree

The degree centrality of a node is the number of edges (connections) to it^26,53^ :

$$ C_{D} \left( v \right) = \mathop \sum \limits_{j} A_{j,v} $$

where A is the adjacency matrix.

#### Betweenness

The betweenness centrality of a node v was introduced in^55^, and it measures the fraction of the shortest paths connecting any two nodes i, and j passing through the node v. In formulas:

$$ C_{B} \left( v \right) = \mathop \sum \limits_{i \ne v \ne j} \frac{{\sigma_{i,j} \left( v \right)}}{{\sigma_{i,j} }} $$

where ${\sigma }_{i,j}$ is the total number of shortest paths connecting i and j, and ${\sigma }_{i,j}\left(v\right)$ the total number of shortest paths connecting *i* and *j* passing through *v*.

#### Closeness

The closeness centrality was introduced in^56^ as the inverse of farness. The closeness of a node *v* is the inverse of the sum of the distance (or shortest path length) between node v and all the other nodes in the graph:

$\tilde{C}_{C} \left( v \right) = \frac{1}{{\mathop \sum \nolimits_{j \ne v} d\left( {j,v} \right)}}$ where $d(j,v)$ is the distance between node j and v.

The definition of closeness centrality we used in this paper is an extension of the one we show above. We implemented the harmonic closeness centrality introduced in^57^, which assumes finite values also in disconnected. This measure is often called harmonic centrality, and it is defined as:

$$ C_{C} \left( v \right) = \mathop \sum \limits_{j \ne v} \frac{1}{{d\left( {j,v} \right)}}. $$

#### Katz

The Katz centrality, introduced in^58^, is defined as:

$$ {\mathbf{C}}_{K} = \alpha {\mathbf{AC}}_{{\mathbf{K}}} + \beta $$

where α is an attenuation factor, A the adjacency matrix, and β a personalization vector. Here, we assume α = 0.01, and β = 1. An alternative definition of Katz centrality is:

$$ {\varvec{C}}_{K} \left( v \right) = \mathop \sum \limits_{{\left\{ {k = 1} \right\}}}^{\infty } \mathop \sum \limits_{{\left\{ {j = 1} \right\}}}^{n} \alpha^{k} \left( {A^{k} } \right)_{vj} + \beta , $$

this second definition makes its interpretation more straightforward: it is counting the number of paths of length *k* connecting the node *v* to each other node *j*, and this value is weighted with the k-power of the attenuation factor $\alpha$.

#### PageRank

PageRank Centrality, introduced in^59^, was the first algorithm used by Google to rank the importance of a webpage. It is defined iteratively by:

$$ {\mathbf{C}}_{{\mathbf{P}}} = \frac{1 - \delta }{N} + \delta \mathop \sum \limits_{{u \in {\Gamma }\left[ v \right]}} \frac{{C_{P} \left( u \right)}}{{C_{D} \left( u \right)}} $$

where $\delta$ is a dumping factor, which we set to 0.85, and $\Gamma \left[v\right]$ is the set of first neighbors of v.

#### BB Index

The BB-index was introduced in^60^, and it is defined as a closeness centrality rescaled by the degree of each node:

$$ C_{BB} \left( v \right) = \mathop \sum \limits_{i \ne v} \frac{{C_{D} \left( v \right)}}{{d\left( {i,v} \right)}} $$

where ${C}_{d}(v)$ is the degree centrality of the node v and $d\left(i,v\right)$ is the distance between nodes i and v.

The BB-Index was introduced to create a network descriptor that combines information about the connectivity of the network and its small-world properties.

### SIR on network

To model the temporal dynamics of the epidemic outbreak, we used a Monte Carlo agent-based Susceptible-Infected-Recovered compartmental model (SIR) to simulate the spread of a disease on real-world networks^9,12,29,61^. In an agent-based SIR, each node of the network represents an individual, which at any given time can be in one of three states, susceptible (S), Infected (I), or Recovered (R). At any time step, a susceptible node is infected by an adjacent infected node with a probability of $\beta$, and every infected node has a probability of $\gamma$ to recover from the infection. In our simulations, we used the values of $\beta$ and $\gamma$ that were recovered from the first months of the COVID pandemic in Italy: $\beta =0.18$, and $\gamma =0.037$^62^. Once an individual is immunized, it cannot be reinfected nor be infective to other individuals/nodes; this can happen if the individual undergoes vaccination or recovers from the infection. Therefore, we initialize the system by setting all the individuals to be susceptible, then we infect one random individual, and finally, we vaccinate a fraction* q* of the susceptible ones accordingly to one of the seven NVS. To simulate the SIR spreading, we parallelized the implementation of the above-mentioned process, realized using the graph-tool Python library^63^. For each set of parameters and real-world network, we performed $1000$ independent simulations where the initially infected node is randomly selected (Table 3).

**Table 3:** NVS summary.

ID	Name	Acronym	Formula	Reference
1	*Random*	RAN	${C}_{R}\left(v\right)=\upxi \in [0, 1]$	^64^
2	*Degree*	DEG	${C}_{D}\left(v\right)={\sum }_{j}{A}_{j,v}$	^55^
3	*Betweenness*	BET	${C}_{B}\left(v\right)={\sum }_{i\ne v\ne j}\frac{{\upsigma }_{i,j}\left(v\right)}{{\upsigma }_{i,j}}$	^55^
4	*Closeness*	CLO	${C}_{C}\left(v\right)={\sum }_{j\ne v}\frac{1}{d\left(j,v\right)}$	^57^
5	*Katz*	KAT	${\mathbf{C}}_{K}=\mathrm{\alpha A}{\mathrm{W}}_{\mathrm{K}}+\upbeta$	^58^
6	*Pagerank*	PAG	${\mathbf{C}}_{\mathbf{P}}=\frac{1-\delta }{N}+\delta \sum_{u\in\Gamma \left[v\right]}\frac{{C}_{P}(u)}{{C}_{D}(u)}$	^59^
7	*BB-Index*	BBI	${C}_{BB}(v)=\sum_{i\ne v}\frac{{C}_{D}(v)}{d(i,j)}$	^60^

For each of the 7 considered NVS we used in this work, we report name, acronym, formula used to calculate, and the reference.

### Spreading indicators (SI) calculation

We considered three **SI**s in this work: the **LCC**, the **TI**, and the **IP**. For every set of simulation parameters $\left(G,q,r,\upgamma ,\upbeta \right)$ where G represents the network, we run the simulation protocol 1000 times, and for each, we calculate the three **SI**s. Finally, we defined **LCC**, TI, and **IP** as the average of the **SI** values over the 1000 iterations.

The largest connected component (**LCC**) is the biggest connected subgraph of the network; here, we consider only its size, namely the number of nodes in it. The total infected (**TI**) is defined as the number of infected individuals at the end of the pandemic event. We obtained it as the difference between the number of Susceptible individuals at time zero and at the end of the simulation: $T{I}_{i}={S}_{i}\left(0\right)-{S}_{i}\left(END\right)$. The Infected Peak (**IP**) is defined as the maximum number of simultaneously infected nodes. Operatively we defined it as the maximum of $I(t)$: ${I}_{i}=\underset{t}{\mathit{max}}{I}_{i}\left(t\right)$. To simplify the comparison of the **SI**s between networks of different sizes, we normalized them by the size of the **LCC** of the initial network (i.e., before any removal).

### Averaged SI calculations

When the average value of a **SI** is used in the text, it is defined in one of the three following ways: $E\left[{\varvec{S}}{\varvec{I}}\right]=\frac{1}{20}{\sum }_{i}{\varvec{S}}{\varvec{I}}(r,q=i,STR,G)$ if it is over the vaccination target *q*; $E\left[{\varvec{S}}{\varvec{I}}\right]=\frac{1}{12}{\sum }_{i}{\varvec{S}}{\varvec{I}}(r,q,STR,G=i)$ if it is averaged over the networks; and $E\left[{\varvec{S}}{\varvec{I}}\right]=\frac{1}{20}\frac{1}{12}{\sum }_{i}{\sum }_{j}{\varvec{S}}{\varvec{I}}(r,q=i,STR,G=j)$ if it is averaged both over networks and over vaccination target.

## Supplementary Information

Supplementary Information.

## Data Availability

The datasets analysed during the current study are available in the ‘Netzschleuder’ repository [https://networks.skewed.de/], in the ‘Stanford Large Network Dataset Collection’ repository [https://snap.stanford.edu/data/index.html], in ‘The Colorado Index of Complex Networks (ICON)’ repository [https://icon.colorado.edu/#!/], and in the ‘SocioPatterns’ repository [http://www.sociopatterns.org/datasets/].
