# Supplementary material for: A comparison of node vaccination strategies to halt SIR epidemic spreading in real-world complex networks
Source: Sci Rep. 2022 Dec 9;12:21355. doi: 10.1038/s41598-022-24652-1 (PMC9734664; doi:10.1038/s41598-022-24652-1)
Supplement: Supplementary file 1 — Supplementary Information. [file 41598_2022_24652_MOESM1_ESM.docx]

# Supplemental Information

**Best strategy frequency for high vaccination targets**

|  | **LCC [%]** | | **TI [%]** | | **IP [%]** | |
| --- | --- | --- | --- | --- | --- | --- |
| **Strategy** | $r=1$ | $r=0.005$ | $r=1$ | $r=0.005$ | $r=1$ | $r=0.005$ |
| RAN | 0 | 0 | 0 | 0 | 0 | 0 |
| DEG | 28 | 66 | 43 | 66 | 43 | 67 |
| BET | 23 | 0 | 19 | 0 | 17 | 0 |
| CLO | 3 | **93** | 8 | **89** | 8 | **89** |
| KAT | 20 | 75 | 33 | 72 | 34 | 72 |
| PAG | **77** | 64 | **66** | 63 | **71** | 64 |
| BBI | 20 | 75 | 30 | 76 | 33 | 75 |

**Table S1:** **Best strategy frequency for high vaccination targets.** In this table we show for the three **SI**s and recalculation step $r=1$ (i.e. no recalculation, non-adaptive NVS), and $r=0.005$ (i.e. semi-adaptive NVS), the percentage of cases where each NVS is among the best strategies, including ties ($\frac{STRAT}{\min\left( SI\left( STRAT \right) \right)}-1<\frac{1}{50})$. The values are obtained considering a fraction of vaccinated population $q>0.5$ across the 12 networks. The bold value in each column indicates the NVS that is more frequently the best for that **SI** and recalculation step r.
